# Inhalations with Brine Solution from the ‘Wieliczka’ Salt Mine Diminish Airway Hyperreactivity and Inflammation in a Murine Model of Non-Atopic Asthma

**DOI:** 10.3390/ijms21134798

**Published:** 2020-07-07

**Authors:** Dominika Zając, Ewelina Russjan, Magdalena Kostrzon, Katarzyna Kaczyńska

**Affiliations:** 1 Department of Respiration Physiology, Mossakowski Medical Research Centre, Polish Academy of Sciences, 02-106 Warsaw, Poland; erussjan@imdik.pan.pl; 2 ‘Wieliczka’ Salt Mine Health Resort, 32-020 Wieliczka, Poland; magdalena.kostrzon@kopalnia.pl

**Keywords:** non-atopic asthma, brine inhalations, add-on therapy, murine model, airway hyperreactivity, airway inflammation

## Abstract

Inhalations with brine solutions are old but underestimated add-ons to pharmacological treatments of inflammatory lung diseases. Although widely used, not all features underlying their action on the respiratory system have been explored. The aim of the present study was to elucidate the mechanism of the beneficial action of inhalations of brine solution from the ‘Wieliczka’ Salt Mine, a Polish health resort, in a murine model of non-atopic asthma. Asthma was induced in BALB/c mice by skin sensitization with dinitrofluorobenzene followed by an intratracheal challenge of cognate hapten. All animals underwent 12 inhalation sessions with brine solution, pure water or physiological saline. Control mice were not inhaled. We found that brine inhalations reduced, as compared to non-inhaled mice, the typical asthma-related symptoms, like airway hyperreactivity (AHR), the infiltration of pro-inflammatory cells into the bronchial tree, and the inflammation of the airways at the level of pro-inflammatory cytokines IL-1α, IL-1β and IL-6. The level of the anti-inflammatory IL-10 was elevated in brine-inhaled mice. Inhalations with pure water increased AHR, whereas saline had no influence, either on AHR or cytokine concentrations. These observations indicate that inhalations with a brine solution from the ‘Wieliczka’ Salt Mine diminish the asthma-related symptoms, mostly by reducing the inflammatory status and by decreasing AHR.

## 1. Introduction

Asthma is a chronic inflammatory disease of the airways. Although its exact causes are not well recognized, several risk factors, including family history, infections in early life, smoking, and environmental pollution could be established. The main features of asthma include respiratory symptoms like wheezing, shortness of breath, cough, and airflow limitation. Depending on the type of immune response, asthma can be divided into allergic (atopic) asthma, related to eosinophilia, and nonallergic (non-atopic) asthma, connected to neutrophilia [1]. Allergic asthma can be induced by allergens, contrary to the nonallergic form evoked by medications, cold, physical effort, and pollution [2,3]. The latter phenotype is more difficult in treatment, demanding higher doses of corticosteroids [4] with more pronounced exacerbations, and longer and more frequent hospitalizations [3,5]. According to the newest recommendations of GINA published in 2019 [6], asthma management should be based on symptom-driven (in case of mild asthma) or daily administered (in the moderate and severe form) inhaled corticosteroids.

Although inhaled medications are safe and almost devoid of systemic side effects, novel therapies, add-on medications and procedures remain under investigation. In the last few years, there has been a tendency to rediscover old, still underestimated treatments. They include, apart from physiotherapy, balneotherapy and climatotherapy, understood as the use of natural sources and climatic conditions in the treatment of various diseases [7,8,9]. Balneotherapy itself uses thermal or mineral waters, and peloids in various therapeutic forms, including baths, drinking, or inhalations [10].

Inhalations are probably one of the oldest therapeutic procedures in respiratory disorders. When performed with physiological saline (0.9% NaCl), they remain a gold standard in treatment of respiratory infections, even in the smallest children [11,12]. They are believed to humidify the airways, to increase the expectoration of the residual mucus, and in general, to facilitate breathing [13,14,15]. Apart from saline and medications, other solutions, like brines and thermal waters from natural sources, are also used for inhalations. Brines themselves are characterized by a high content of chloride and sodium ions, whereas other natural thermal waters contain a significant number of other ions and solutes, like carbon dioxide, bicarbonates, and sulfides. Depending on the mineral composition and concentration of the respective ions in the thermal water or brine, they show various beneficial effects, including mucolysis [16,17], diminution of inflammation [18,19], improvement of asthma control [20], decrease in COPD exacerbations [21,22], reduction of asthma- and allergy-related symptoms [23,24,25,26], and stimulation of the immune system [27]. Inhalations with thermal waters and brines are often performed under the control of medical professionals in rehabilitation centers and health resorts, as part of a health-promoting and/or therapeutic stay. An example of a health resort which specializes in the treatment of respiratory disorders is the Health Resort of the ‘Wieliczka’ Salt Mine in Wieliczka, Poland. Located in one of the oldest salt mines in Europe (dated to the 13th century), it offers unique therapeutic opportunities, including subterraneotherapy (climatotherapy in underground caves), respiratory rehabilitation in a germ- and pollution-free environment, and a full set of rehabilitation procedures of a health resort with the use of the local salt-based resources [28]. Indications of such a stay are recurrent and chronic bronchitis, various forms of rhinitis and rhinosinusitis, stable asthma, cystic fibrosis, COPD, and respiratory forms of allergies [29]. The beneficial effects of such a treatment seem to be long-lasting. However, the mechanisms underlying this action remain unclear.

Therefore, the aim of this study was to determine whether a cycle of inhalations with brine-containing thermal water would influence the airway hyperreactivity and inflammatory markers in non-atopic asthma subjects. For this reason, a murine model of hapten-induced non-atopic asthma was used. The brine originated from the ‘Wieliczka’ Salt Mine (outflow W-VII-16, depth 255 m underground). It is of sodium chloride character, and as the main therapeutic solution for inhalations in the rehabilitation center of the health resort, it is diluted to 2%. Some of the results presented here were published in the form of a conference abstract [30].

## 2. Results

The term “sham-sensitized” animals refers to subjects which underwent the procedure of sensitization only with the vehicle (thus, showing no symptoms of asthma) and were inhaled with saline in the same scheme as other groups. As saline is believed to have no influence on respiration in healthy subjects, the group (VEH+S) is set herein as the control group. Parallel, sensitized but not inhaled animals (DNFB) were set as the second control group, as they did not undergo any additional treatment.

### 2.1. Basal Ventilatory Parameters

Basal ventilatory parameters before and after the set of inhalations are presented in Table 1. The course of inhalations did not significantly change the breathing frequency or the inspiratory and expiratory times in any of the groups. Only an increase in tidal volume was observed in the sham-sensitized saline-inhaled (VEH+S) group, together with an increase in minute ventilation in the VEH+S and sensitized brine-inhaled (DNFB+B) groups. Additionally, the enhanced pause (Penh) measured for the saline provocation before methacholine was significantly extended after the second DNS challenge in the case of the sensitized but non-inhaled (DNFB) group. At the same time, only tidal volume and minute ventilation after the series of inhalation were significantly higher in the sensitized saline-inhaled (DNFB+S) group, as compared to the non-inhaled DNFB one. Other basal ventilatory parameters remained invariable between the groups after the course of inhalations, as well as within the groups before and after the treatment.

### 2.2. Airway Hyperreactivity (AHR) to Methacholine (MCh)

**Figure 1:**
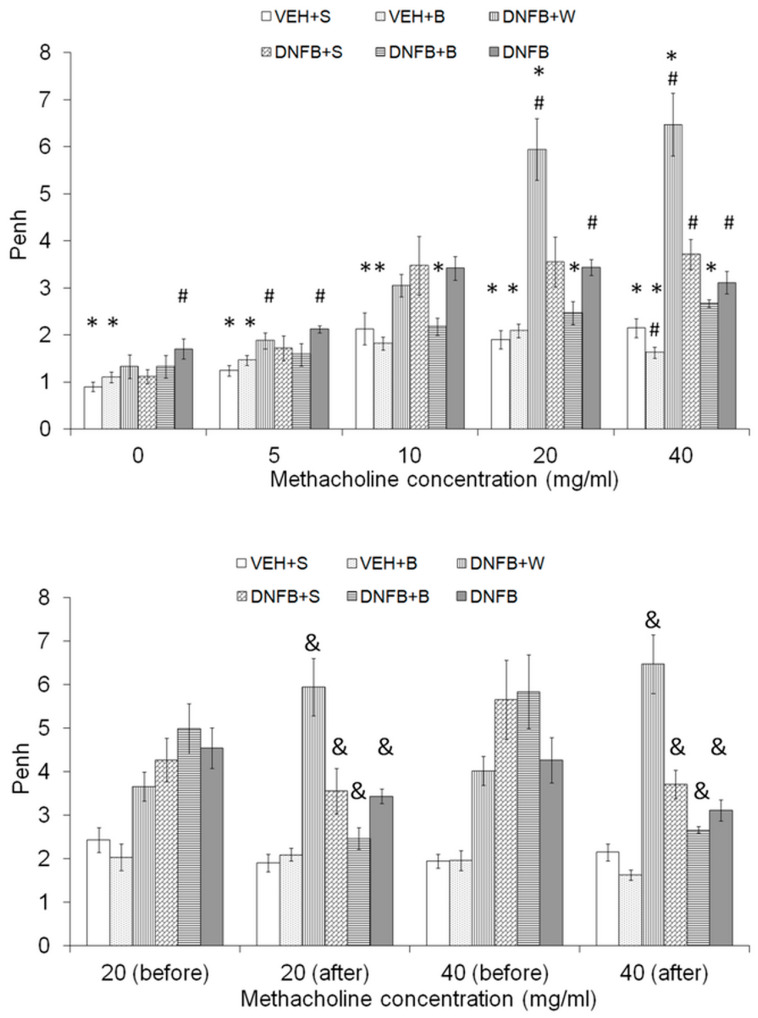
**Upper panel:** Airway hyperreactivity to methacholine. # *p* < 0.05 vs. VEH+S (Mann–Whitney U test); * *p* < 0.05 vs. DNFB (Mann–Whitney U test); means ± SE, *n* = 5–10. **Lower panel:** Airway hyperreactivity for the concentration of methacholine 20 and 40 mg/mL before and after inhalations. & *p* < 0.05 before vs. after inhalation (Wilcoxon test); means ± SE, *n* = 5–10. VEH+S, sham-sensitized saline-inhaled; VEH+B, sham-sensitized brine-inhaled; DNFB+W, sensitized pure water inhaled; DNFB+S, sensitized saline inhaled; DNFB+B, sensitized brine inhaled; DNFB, sensitized non-inhaled.

Airway hyperreactivity was assessed by measurements of Penh during a methacholine (MCh) test. There were changes in the profile of the MCh test within the groups before and after the course of inhalations (Figure 1, lower panel). In case of the DNFB+W group, Penh was increased after water inhalations and nebulization of MCh in concentrations over 10 mg/mL. It was significantly lower after inhalations of all MCh concentrations in case of the DNFB+S and DNFB+B. In case of the DNFB group, Penh was significantly lower after the second DNS challenge for the provocation with MCh concentrations over 10 mg/mL. No changes in the MCh test course before and after inhalations could be observed for the VEH+S and VEH+B groups.

The course of Penh changes after 14 days in all groups is presented in Figure 1 (upper panel). Briefly, in sham-sensitized animals (VEH+S, VEH+B), the reaction to the methacholine stimulus was weak. In the case of sensitized pure water-inhaled (DNFB+W) animals, AHR increased from the MCh concentration of 10 mg/mL and remained elevated for the entire course, showing, at the same time, the highest values of Penh. In sensitized, saline-inhaled (DNFB+S) subjects, the AHR increased after the 5 mg/mL of stimulus and stayed on the same level from the concentration of 10 mg/mL. Sensitized brine-inhaled (DNFB+B) mice showed no change in AHR, despite the increasing concentration of MCh. The same was observed for sham-sensitized subjects (VEH+S, VEH+B). There were no significant differences between the DNFB+B and VEH (VEH+S, VEH+B) groups. The course of AHR in response to increasing MCh concentrations in DNFB sensitized but non-inhaled animals was similar to the one observed for DNFB+S subjects.

### 2.3. Cell Infiltration

**Figure 2:**
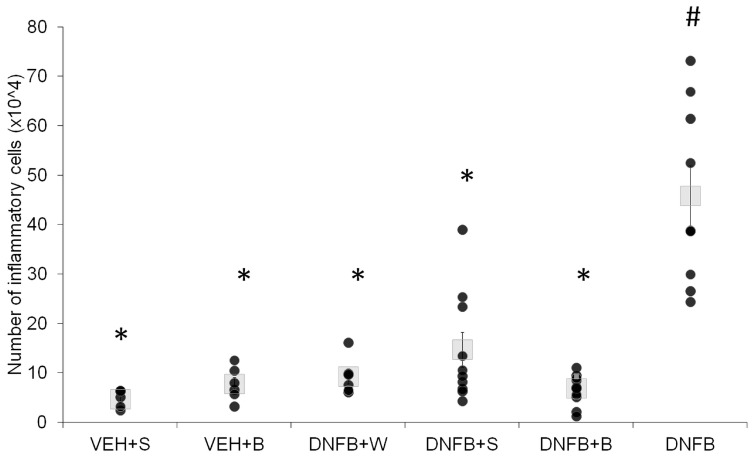
Total number of inflammatory cells per lung. # *p* < 0.05 vs. VEH+S (Mann–Whitney U test); * *p* < 0.05 vs. DNFB (Mann–Whitney U test); means ± SE, *n* = 6–10; VEH+S, sham-sensitized saline-inhaled; VEH+B, sham-sensitized brine-inhaled; DNFB+W, sensitized pure water inhaled; DNFB+S, sensitized saline inhaled; DNFB+B, sensitized brine inhaled; DNFB, sensitized non-inhaled.

**Figure 3:**
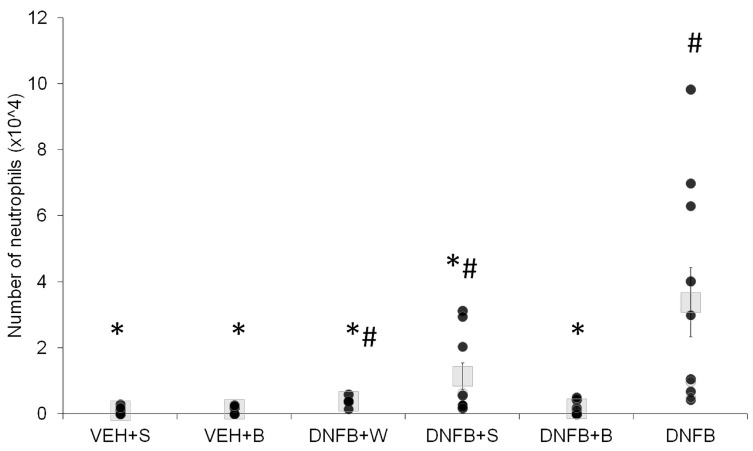
Total number of neutrophils per lung. # *p* < 0.05 vs. VEH+S (Mann–Whitney U test); * *p* < 0.05 vs. DNFB (Mann–Whitney U test); means ± SE, *n* = 6–10 VEH+S, sham-sensitized saline-inhaled; VEH+B, sham-sensitized brine-inhaled; DNFB+W, sensitized pure water inhaled; DNFB+S, sensitized saline inhaled; DNFB+B, sensitized brine inhaled; DNFB, sensitized non-inhaled.

The number of total inflammatory cells and neutrophils is presented in Figure 2 and Figure 3.

Sensitization itself provoked an increase in the total number of inflammatory cells, as it could be observed in the DNFB group. Additionally, the number of neutrophils was strongly increased in this group. The population of total inflammatory cells consisted mostly of macrophages (91%), neutrophils (8%) and a small number of lymphocytes (less than 1%). No eosinophils or basophils could be found in any of the studied groups. Every kind of inhalation significantly decreased the total number of cell infiltrate. Inhalations with pure water (DNFB+W) and saline (DNFB+S) decreased this number; however, these levels were still higher than those of sham-sensitized (VEH+S) animals. Brine inhalations (DNFB+B) decreased the total number of inflammatory cells and neutrophils to levels comparable to the sham-sensitized groups.

### 2.4. Airway Inflammation

**Figure 4:**
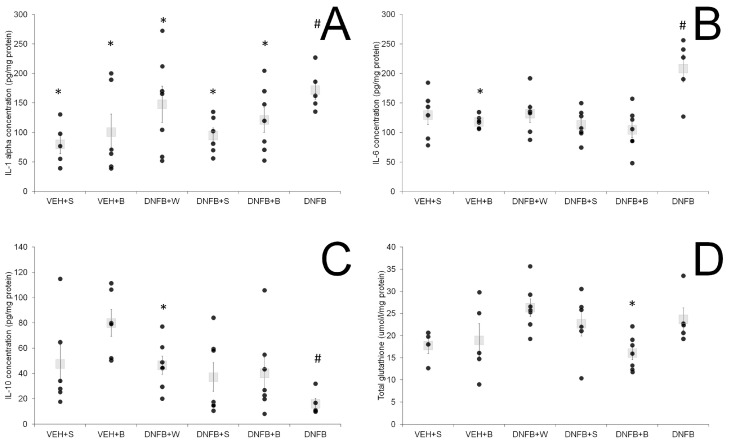
IL-1α (**A**), IL-6 (**B**), IL-10 (**C**) and total glutathione (**D**) levels in lung tissue. # *p* < 0.05 vs. VEH+S (Mann–Whitney U test); * *p* < 0.05 vs. DNFB (Mann–Whitney U test); means ± SE, *n* = 5–7 VEH+S, sham-sensitized saline-inhaled; VEH+B, sham-sensitized brine-inhaled; DNFB+W, sensitized pure water inhaled; DNFB+S, sensitized saline inhaled; DNFB+B, sensitized brine inhaled; DNFB, sensitized non-inhaled.

**Figure 5:**
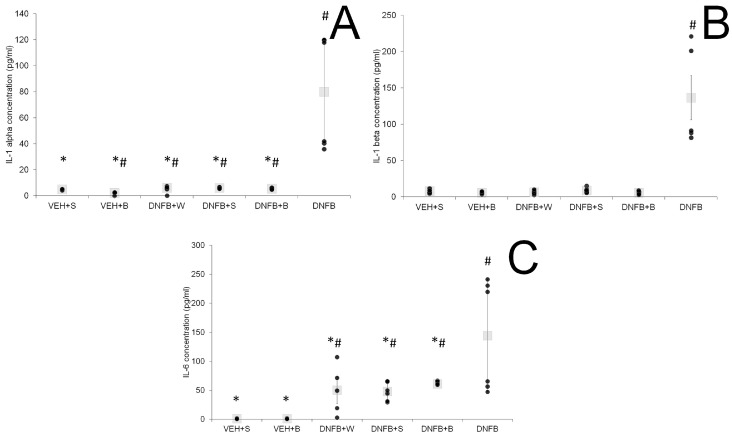
IL-1α (**A**), IL-1β (**B**) and IL-6 (**C**) levels in BALF. # *p* < 0.05 vs. VEH+S (Mann–Whitney U test); * *p* < 0.05 vs. DNFB (Mann–Whitney U test); means ± SE, *n* = 5–8; VEH+S, sham-sensitized saline-inhaled; VEH+B, sham-sensitized brine-inhaled; DNFB+W, sensitized pure water inhaled; DNFB+S, sensitized saline inhaled; DNFB+B, sensitized brine inhaled; DNFB, sensitized non-inhaled.

The levels of cytokines in lung tissue and BALF are presented in Figure 4A–C and Figure 5A–C, respectively. In lung tissue, levels of pro-inflammatory cytokines IL-1α and IL-6 were significantly elevated in the DNFB group. A decrease in concentration was observed in all sensitized and inhaled groups (DNFB+W, DNFB+S, DNFB+B) to levels comparable to the ones displayed in the sham-sensitized ones (VEH+S and VEH+B). In case of the anti-inflammatory IL-10, the lowest level was detected in the DNFB group, whereas in the VEH+S and VEH+B group, the highest levels were detected. There was no difference in concentration between all sensitized and inhaled groups compared to the sham-sensitized one.

In BALF, the levels of pro-inflammatory cytokines IL-1α and IL-6 were elevated in the case of DNFB animals. Any type of inhalations decreased these levels, however, not to values observed for sham-sensitized animals. IL-1β was strongly increased in DNFB animals, whereas in all sensitized and inhaled groups, the levels of this cytokine remained comparable to the sham-sensitized one.

### 2.5. Oxidative Stress Marker

Total glutathione levels as markers of oxidative stress are presented in Figure 4D. The untreated (non-inhaled) DNFB mice showed a slightly elevated (however insignificantly) level of total glutathione as compared to the control VEH+S and VEH+B groups. Inhalations with water (DNFB+W) or physiological saline (DNFB+S) did not lower these levels, in contrary to inhalations with brine (DNFB+B), where the total glutathione was significantly decreased compared to the non-inhaled (DNFB) animals.

## 3. Discussion

In the present study, we investigated whether a course of inhalations with brine solution would influence non-atopic asthma symptoms in mice. For this reason, we measured airflow limitation and bronchospasm described by the enhanced pause, and parameters of respiratory pattern before and after the therapeutic procedure, both in sensitized animals (showing symptoms of asthma) and sham-sensitized (without symptoms of the disease, healthy) subjects. Additionally, we measured the cellular influx into the airways, as well as the levels of inflammatory cytokines and the redox status, all of them being hallmarks of asthma. They all will be discussed in short thereinafter. At this point, we assumed that the repeated instillation of DNS evokes at least similar asthma symptoms to those observed after the first DNS challenge, enabling us to describe the course of a recurrence of the disease after a certain time, as well as the influence of an intervention.

First, we showed that brine inhalations in sham subjects had no influence on respiratory parameters, airway hyperreactivity to methacholine (AHR) or inflammatory markers in lung tissue and BALF, as compared to sham-sensitized, saline-inhaled (control) animals, having in mind that hypertonic solution can potentially irritate the airways [31]. The situation changed dramatically when we took under consideration sensitized mice inhaled with pure water, physiological saline and 2% brine solution. We found that inhalations with the latter one nearby totally suppressed the response to methacholine comparable to the levels observed for sham-sensitized animals. This is in line with findings of Tanizaki et al. [32], who found that inhalations with thermal water ameliorate lung function and decrease airway hyperreactivity in severe and difficult-to-treat asthmatics. It is generally accepted that inhalations with hyperosmolar ionic solutions enhance pulmonary function in respiratory diseases [14,33,34], and facilitate mucociliary clearance [35]. Interestingly, we verified that inhalations with saline did not change airway hyperreactivity.

At the same time, hypo-osmolar solutions, as the here used pure water, have been shown to provoke bronchoconstriction [36,37,38], and increase the number of exacerbations of asthma [39]. When applied in a repeated manner, pure water can potentially lead to functional changes and the injury of the bronchial epithelium [37], without stimulating the inflammatory cells to release inflammatory cytokines [36]. These observations were confirmed in our study, as pure water-inhaled sensitized animals showed a much greater increase in airway responsiveness to methacholine, and did not have more significant symptoms of inflammation than non-inhaled, sensitized with DNFB animals. It has been demonstrated previously that hyperreactive bronchoconstriction can be induced without an inflammatory immune response [40].

Another interesting finding was the lack of differences in the basal ventilatory parameters before methacholine stimuli between sensitized and sham-sensitized animals prior to the inhalations. This confirmed the results of Vanoirbeek et al. [41], who could not find significant differences in respiratory parameters in OVA-albumin and chemical (TDI)-induced asthmas, before and after sensitization. In our study, only a set of inhalations with brine in sensitized animals (DNFB+B) increased significantly basal minute ventilation comparably to the one in sham-sensitized, saline-inhaled subjects (VEH+S). This could once again speak for the beneficial effects of brine inhalations.

Apart from airway hyperreactivity, another feature of asthma is the airway inflammation described by the influx of inflammatory cells into the airways and the levels of inflammatory cytokines in both the lung tissue and BALF. The total number of inflammatory cells in sensitized, non-inhaled animals in our study was about three to ten times higher than in previous reports [42,43], which is probably in relation to the repeated hapten challenge. Moreover, we obtained a comparably elevated level of neutrophils, together with a lack of eosinophils, as it was observed previously [42,43]. Neutrophilia and the absence of eosinophils are key features of non-atopic asthma [44], both in humans and in animal models [45], which may determine the cytokine spectrum in blood, BALF, and lung tissue [1]. Here, the number of both total inflammatory cells and neutrophils did not vary between the brine-inhaled (DNFB+B) and sham-sensitized (VEH+S and VEH+B) groups. This suggests a strong reduction of inflammation at the level of the epithelial layer of the airways, crucial in the pathogenesis of asthma [46].

We were not able to find any significant differences between the sensitized inhaled and the sham-sensitized groups in the case of lung tissue pro-inflammatory IL-1α, IL-1 β and IL-6, while in the non-inhaled sensitized group, they remained elevated. However, the anti-inflammatory cytokine IL-10 was elevated in all inhaled groups compared to the sensitized non-inhaled one. In regard to the findings of Boshtam et al. [47], this speaks for a prolonged inhibition of pro-inflammatory mediators like IL-1α and IL-6. Additionally, the analysis of BALF showed an anti-inflammatory action of brine inhalations at the level of IL-1α and IL-1β, as also described by Zaripova et al. [48]. All inhalations also decreased the levels of IL-6 in lung tissue and BALF, cytokine released by macrophages, found in the sputum of asthmatics and associated with asthma airway inflammation [49].

The question arises as to what exactly induces the advantageous actions of brine inhalations on cellular influx and AHR to metacholine. According to Berend et al. [2], airway hyperreactivity depends not only on inflammatory mediators like IL-1β, which is believed to induce AHR [50,51], but also on the presence of inflammatory cells and epithelial damage. Therefore, the plausible mechanism responsible for AHR reduction observed in our study could be related to alleviated inflammation: a decrease in IL-1β, IL-1α and IL-6 concentrations, responsible for a decreased influx of inflammatory cells. IL-6 possibility to recruit neutrophils in acute inflammation and monocytes in chronic one has been previously described [52]. IL-1β challenge into rat trachea accumulated neutrophils in BALF and produced increased airway responsiveness [51]. In fact, both cytokines, IL-1β and IL-1α, reduced in our study by brine solution, were displayed to play a significant role in AHR and inflammatory cells influx; their blockade reduced the phenotype of murine asthma [53]. Additional information could provide an investigation of a wider range of inflammatory cytokines, including those engaged in neutrophil chemoattraction, such as IL-17 or IL-8, and this is planned in our future experiments [54].

Hyperresponsiveness to methacholine has been demonstrated to be also associated with a decreased expression of aquaporins (AQP), the major water channels in the bronchial and alveolar epithelium [55]. Since hyper-osmolar stress induces the expression of AQP [56], we can speculate that inhalations with brine solution could possibly interfere with the AQP system and affect AHR in some way. Parallel, hyperosmotic solutions, such as brines, open tight junctions, and as a consequence, increase the transepithelial water transport [57]. Additionally, they break ionic bounds within the mucus and thus lower its viscosity [57,58] and in consequence, lead to an increase in mucociliary clearance [34], and facilitate breathing.

Another feature of asthma is chronic oxidative stress and the imbalance of the redox status [59,60], pronounced, among others, by an elevated level of glutathione [61], the accumulation of its oxidized and the reduction of its reduced form [62], leading to an increased susceptibility to lung injury [60]. Brine inhalations decreased the levels of total glutathione in sensitized animals. This suggests that the beneficial influence of brine inhalations might be related to a correction of the redox imbalance via the glutathione system.

It is worth noticing that hypertonic solutions act mostly locally on the epithelium of the airways, and are able to enhance the resolution of alveolar edema, one of the symptoms of asthma attacks [63]. They also act in an anti-inflammatory manner by disrupting cytokine signaling [64], as was the case in our study. However, the main issue about inhalations of hyperosmotic solutions in asthma is the double action. On one hand, they can induce bronchoconstriction, as described by Schoeffel et al. [37], probably by a mast cell- and histamine-dependent mechanism, and on the other, inhalations with these solutions are beneficial in various respiratory disorders [13,14,24,65,66,67]. One of the possible explanations is the stage of the disease. During acute exacerbations, a bronchoconstriction evoked by various factors including hyperosmolar stimuli [37] seems to be more probable, and as a consequence, any additional irritant procedure is rather inadvisable. During well-controlled, relatively symptom-free periods, hyperosmolar treatments may remain safe and develop their above-mentioned beneficial effects.

Taken together, the beneficial effect observed after a set of brine inhalations includes a decrease of AHR to methacholine, the reduction of cellular influx, at least partial anti-inflammatory action and the correction of the redox imbalance.

Apart from osmolality, which was, in the present study, described by the use of pure water, physiological saline (0.9%) and brine solution (2%), the composition of the inhaled solution also plays a crucial role. There are several types of thermal waters divided according to the composition and described in detail elsewhere [68,69]. In the present study, brine, a thermal water with a prevalence of sodium and chloride ions, was used. However, other ions, namely magnesium and calcium, are also important constituents of the solution, with magnesium probably being the most important one. Costantino et al. [25] points out the importance of magnesium ions in the antioxidant action of the inhaled brine solution. Magnesium itself reduces oxidative stress and protects against inflammation [70]. The serum content of this mineral is decreased in asthmatics [71,72], and is associated with low lung volume and airflows [73]. Magnesium inhalations resolve bronchoconstriction [74,75], and ameliorate the asthma severity score [76]. Thus, one might say that brine inhalations were some kind of weak supplementation of magnesium directly to the mouse’s airways, and the observed beneficial effect of this treatment might also be partially related to the magnesium content of the inhaled brine.

In the present study, we used the enhanced pause (Penh) as a tool for the determination of airway hyperreactivity and bronchial spasm. We are aware that this parameter is controversial and has both its supporters [77,78,79] and opponents [80,81,82]. However, the main aim of this study was the description of airway hyperreactivity as a repeated measure in the same subject before and after period of inhalations and up to now, this is possible only with the use of whole body plethysmography (WBP) [83]. There are several requirements under which Penh correlates to airway resistance and compliance. The most important is that a correct strain of mice has to be used. Adler et al. [84], as well as Duguet et al. [85], showed that Penh and airway resistance/compliance are comparable and correlated in BALB/c, but not in C57BL/6 mice, thus, the BALB/c mice were used in our study.

Inhalations with brine solutions are widely used, not only at home, but also, and even first of all, in rehabilitation centers of health resorts, as one of the most important rehabilitation procedures in respiratory disorders. Contrary to physiotherapy, whose mechanisms of action are well known and described, the mode of action of inhalations is only partially elaborated in the case of difficult-to-treat asthma. Here we showed that in non-atopic asthma model, one of the most difficult in treatment, inhalations with brine solution decrease airway inflammation, and decrease airway hyperreactivity to methacholine. This might, at least partially, explain the successful use of brine inhalations in various health resorts as a recognized therapeutic procedure. According to Polish Law, inhalations as a rehabilitation treatment prescribed by a licensed physician are, under certain conditions, paid by the insurance.

Taken together, all kinds of inhalations showed more or less pronounced beneficial effects, affecting the various features of asthma. Out of all applied solutions, the 2% brine solution had the most positive influence on the airway hyperreactivity and inflammation.

## 4. Materials and Methods

All experiments were conducted in accordance with the European Directive 2010/63/EU on Animal Experimentation and approved by the I Local Ethics Committee in Warsaw, Poland (permit n. 396/2017 obtained on the 24 October 2017).

### 4.1. Drugs and Reagents Used in the Study

The reagents and drugs used in the study were obtained from: Sigma-Aldrich (Poznań, Poland): 1-fluoro-2, 4-dinitrobenzene (DNFB), dinitrobenzene sulfonic acid (DNS), olive oil, acetone, protease inhibitor cocktail, and Triton X-100; Biowet (Puławy, Poland): ketamine, xylazine, and pentobarbitone; Polpharma (Warsaw, Poland): pure water (Aqua pro injectione) and physiological saline (Injectio natrii chlorati isotonica); ‘Wieliczka’ Salt Mine (Wieliczka, Poland): brine (outflow W-VII-16, depth 255 m underground) of the following composition (mg/L): Cl^−^ 38107.0, SO_4_^2−^ 2771.1, HCO_3_^−^ 717.4, Na^+^ 26359.5, Ca^2+^ 673.7, Mg^2+^ 235.8, K^+^ 52.7, H_2_SiO_3_ 20.7, HBO_2_ 22.65, total mineralization 68.9 g/L; Analab (Warszawa, Poland): Türk reagent, Hemastain dye; R&D Systems, Inc. (Minneapolis, MN, USA): ELISA kits for IL-1α, IL-1β, IL-6, and IL-10; Cayman Chemical (Ann Arbor, MI, USA): ELISA kit for glutathione.

### 4.2. Experimental Procedures

#### 4.2.1. Sensitization and Methacholine Challenge

A mouse model of non-atopic asthma characterized by neutrophilia previously described [86,87], with slight modifications, was utilized. Briefly, 50 male mice aged 7–8 weeks, weighing 25–30 g were used in the study, and were randomly divided into 6 groups. On day one, the animals were skin-sensitized with 0.5% DNFB (1-fluoro-2, 4-dinitrobenzene) in vehicle (acetone: olive oil 4:1 *v*/*v*) (sensitized groups) or vehicle alone (sham-sensitized groups) on shaved thorax (50 μL) and four paws (50 μL) under light ketamine/xylazine (70 mg/kg + 10 mg/kg) anesthesia. One day later, the sensitization on the thorax was repeated. DNFB is a small-molecular-weight compound-hapten, that acquires immunogenicity after attachment to the protein carrier [88]. On day 6, the animals were intratracheally challenged with cognate hapten-50 μL of 0.6% DNS (dinitrobenzene sulfonic acid) dissolved in pure water. Then, 24 h later, all animals underwent a methacholine (MCh) test in a plethysmographic chamber (Buxco Electronics, Inc., Wilmington, NC, USA). After a habituation time of 10 min, 10 min inhalations with increasing concentrations of nebulized MCh (0, 5, 10, 20 and 40 mg/mL, dissolved in saline), each followed by 10 min of room air breathing, were performed, and continuously recorded using the Buxco software. The following parameters were taken under consideration for further analyses: breathing frequency, tidal volume, minute ventilation, inspiratory and expiratory time, and enhanced pause (Penh).

#### 4.2.2. Inhalations

**Figure 6:**
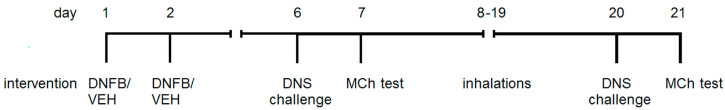
The outline of the experiment. DNFB, skin sensitization with 1-fluoro-2, 4-dinitrobenzene; VEH, skin sensitization with vehicle (acetone: olive oil 4:1 *v*/*v*); DNS, intratracheal challenge with dinitrobenzene sulfonic acid; MCh, test with increasing concentrations of metacholine.

From the 8th day on, both the DNFB-sensitized (sensitized) and vehicle-sensitized (sham-sensitized) animals underwent a 12-day inhalation cycle with an aerosol of pure water (only sensitized animals) (DNFB+W), saline-0.9% NaCl (sensitized and sham-sensitized animals) (DNFB+S and VEH+S, respectively), or 2% brine from the ‘Wieliczka’ Salt Mine (sensitized and sham-sensitized animals) (DNFB+B and VEH+B, respectively). Each inhalation lasted 15 min and was preceded with a 5-min habituation time. The volume of the nebulized solution was 0.45 mL. One group of DNFB-sensitized animals remained non-inhaled (DNFB). One day after the last inhalation, all animals from all groups were challenged once again with DNS. Then, 24 h later, the methacholine test was repeated as described above. An outline of the entire experiment is presented in Figure 6.

#### 4.2.3. BALF Collection and Cytological Analysis

After the last MCh test, the mice were sacrificed with an intraperitoneal injection of pentobarbitone (50 mg/kg). The trachea was cannulated and 1 mL of PBS with a protease inhibitor cocktail was injected and withdrawn from the lungs to obtain the broncho-alveolar lavage fluid (BALF). An additional 3 × 1 mL of PBS were flushed and pooled. All samples were centrifuged at 160× *g* (1500 rpm) for 10 min at 4 °C, and the BALF cells were isolated. The supernatant from the 1st lavage was frozen at −80 °C for further examinations of inflammatory markers. Isolated cells were re-suspended in 150 μL of saline. Fifty microliters of the suspension were diluted with 100 μL of the Türk reagent and counted in a Bürker–Türk chamber, whereas the remaining 100 μL of the suspensions were cytocentrifuged on microscopic slides at 700 rpm for 10 min, using a Shandon cytospin (Thermo Shanon, Cambridge, UK), air-dried, and stained with hemastain dye (a fast staining kit based on eosin and azure solutions). A differential cell count on 500 cells from each sample was performed under a light microscope. Results are expressed as the number of mononuclear cells (macrophages and leukocytes) and neutrophils per lung.

#### 4.2.4. Lung Collection and Homogenization

The lungs were isolated and frozen at −80 °C for further determinations of inflammatory markers. The supernatants of lung homogenates were obtained by homogenization under liquid nitrogen in PBS/protease inhibitor cocktail/Triton X-100, followed by a centrifugation at 15,000× *g* (14,000 rpm) for 10 min at 4 °C.

#### 4.2.5. Biochemical Analyses

Cytokines and glutathione in BALF and lung supernatants were analyzed using ELISA kits, according to the manufacturers’ instruction. The ELISA plates were read using Epoch ELISA reader (BioTek Instruments, Winooski, VT, USA). Concentrations of cytokines were expressed as pg/mg of total protein in the case of lung tissue homogenates and pg/mL in the case of BALF; total glutathione in lung tissue was expressed as µmol/mg of total protein.

### 4.3. Statistical Analyses

All values are expressed as means ± standard error. Differences between the groups were analyzed with the use of the Mann–Whitney U-test, whereas those within the group described the differences between the state before and after the treatment, with the Wilcoxon test. All statistical calculations were performed on the commercially available STATISTICA 12 software. *p* < 0.05 was considered to be statistically significant.

## Figures and Tables

**Table 1:** Basal ventilatory parameters before (day 7) and after (day 21) inhalations (group sensitized but non-inhaled (DNFB) was not inhaled).

		f (1/min)	TV (mL)	MV (mL/min)	Ti (s)	Te (s)	Penh
VEH+S	Before (day 7)	457.09 ± 30.07	0.317 ± 0.018	142.06 ± 12.91	0.0491 ± 0.0016	0.1386 ± 0.0294	1.258 ± 0.176
	After (day 21)	468.92 ± 15.76	0.367 ± 0.010 #	167.34 ± 7.66 #	0.0511 ± 0.0010	0.1194 ± 0.0090	0.898 ± 0.099
VEH+B	Before (day 7)	484.54 ± 16.86	0.375 ± 0.030	175.20 ± 15.47	0.0513 ± 0.0018	0.0998 ± 0.0118	0.997 ± 0.124
	After (day 21)	481.53 ± 8.11	0.348 ± 0.020	164.13 ± 10.79	0.0489 ± 0.0007	0.1239 ± 0.0121	1.098 ± 0.112
DNFB+W	Before (day 7)	407.09 ± 41.93	0.359 ± 0.040	202.14 ± 20.32	0.0608 ± 0.0067	0.1219 ± 0.0200	1.537 ± 0.334
	After (day 21)	448.06 ± 12.45	0.429 ± 0.037	187.59 ± 15.81	0.0561 ± 0.0029	0.1116 ± 0.0115	1.327 ± 0.256
DNFB+S	Before (day 7)	478.83 ± 13.48	0.354 ± 0.035	165.22 ± 14.26	0.0505 ± 0.0023	0.1081 ± 0.0075	1.242 ± 0.171
	After (day 21)	467.04 ± 9.77	0.421 ± 0.028 *	191.19 ± 11.65 *	0.0530 ± 0.0016	0.1082 ± 0.0104 *	1.111 ± 0.149
DNFB+B	Before (day 7)	428.31 ± 29.07	0.307 ± 0.018	121.03 ± 10.13	0.0516 ± 0.0031	0.1694 ± 0.0305	1.479 ± 0.167
	After (day 21)	468.37 ± 11.76	0.364 ± 0.021	165.85 ± 9.41 #	0.0504 ± 0.0012	0.1310 ± 0.0246	1.322 ± 0.240
DNFB	Before (day 7)	459.94 ± 16.20	0.342 ± 0.013	149.50 ± 8.08	0.0509 ± 0.0016	0.1273 ± 0.0196	1.181 ± 0.118
	After (day 21)	472.73 ± 9.80	0.344 ± 0.017	154.35 ± 8.18	0.0506 ± 0.0012	0.1388 ± 0.0134	1.702 ± 0.212 #

* *p* < 0.05 vs. DNFB (Mann–Whitney U test); # *p* < 0.05 before vs. after inhalations (Wilcoxon test); means ± SE, *n* = 5–10. VEH+S, sham-sensitized saline-inhaled; VEH+B, sham-sensitized brine-inhaled; DNFB+W, sensitized pure water inhaled; DNFB+S, sensitized saline inhaled; DNFB+B, sensitized brine inhaled; DNFB, sensitized non-inhaled; f, breathing frequency; TV, tidal volume; MV, minute ventilation; Ti, inspiratory time; Te, expiratory time; Penh, enhanced pause.

## Abbreviations

DNFB	1-fluoro-2, 4-dinitrobenzene (as substance)
DNS	dinitrobenzene sulfonic acid
MCh	methacholine
BALF	broncho-alveolar lavage fluid
DNFB+W	sensitized pure water inhaled animals
DNFB+S	sensitized saline inhaled animals
DNFB+B	sensitized brine inhaled animals
VEH+S	sham-sensitized saline-inhaled animals
VEH+B	sham-sensitized brine-inhaled animals
DNFB	sensitized non-inhaled animals (as experimental group)

## References

[1] Vizmanos-Lamotte G., Moreno-Galdo A., Munoz X., Gomez-Olles S., Gartner S., Cruz M.J. (2013). Induced sputum cell count and cytokine profile in atopic and non-atopic children with asthma. Pediatr. Pulmonol..

[2] Berend N., Salome C.M., King G.G. (2008). Mechanisms of airway hyperresponsiveness in asthma. Respirology.

[3] Esteban-Gorgojo I., Antolín-Amérigo D., Domínguez-Ortega J., Quirce S. (2018). Non-eosinophilic asthma: Current perspectives. J. Asthma Allergy.

[4] Green R.H., Brightling C.E., Woltmann G., Parker D., Wardlaw A.J., Pavord I.D. (2002). Analysis of induced sputum in adults with asthma: Identification of subgroup with isolated sputum neutrophilia and poor response to inhaled corticosteroids. Thorax.

[5] Turkeli A., Yilmaz O., Taneli F., Horasan G.D., Kanik E.T., Kizilkaya M., Gozukara C., Yuksel H. (2015). IL-5, IL-8 and MMP -9 levels in exhaled breath condensate of atopic and nonatopic asthmatic children. Respir. Med..

[6] GINA Global Initiative for Asthma; Global Strategy for Asthma Management and Prevention. www.ginasthma.org.

[7] Eberlein B., Huss-Marp J., Pfab F., Fischer R., Franz R., Schlich M., Leibl M., Allertseder V., Liptak J., Kriegisch M. (2014). Influence of alpine mountain climate of Bavaria on patients with atopic diseases: Studies at the Environmental Research Station Schneefernerhaus (UFS—Zugspitze)—A pilot study. Clin. Transl. Allergy.

[8] Gaisberger M., Šanović R., Dobias H., Kolarž P., Moder A., Thalhamer J., Selimović A., Huttegger I., Ritter M., Hartl A. (2012). Effects of ionized waterfall aerosol on pediatric allergic asthma. J. Asthma.

[9] Menger W., Schellhaas J. (1980). A telemetric study of the secretolytic effect of sea-air on children with bronchial asthma (author’s transl). Prax Klin Pneumol..

[10] Gutenbrunner C., Bender T., Cantista P., Karagülle Z. (2010). A proposal for a worldwide definition of health resort medicine, balneology, medical hydrology and climatology. Int. J. Biometeorol..

[11] Tomooka L.T., Murphy C., Davidson T.M. (2000). Clinical study and literature review of nasal irrigation. Laryngoscope.

[12] Wei C.C., Adappa N.D., Cohen N.A. (2013). Use of topical nasal therapies in the management of chronic rhinosinusitis. Laryngoscope.

[13] Donaldson S.H., Bennett W.D., Zeman K.L., Knowles M.R., Tarran R., Boucher R.C. (2006). Mucus clearance and lung function in cystic fibrosis with hypertonic saline. N. Engl. J. Med..

[14] Elkins M.R., Robinson M., Rose B.R., Harbour C., Moriarty C.P., Marks G.B., Belousova E.G., Xuan W., Bye P.T.P. (2006). A controlled trial of long-term inhaled hypertonic saline in patients with cystic fibrosis. N. Engl. J. Med..

[15] Rassulova M.A., Razumov A.N., Aĭrapetova N.S. (2007). Use of natural physical factors in the rehabilitative treatment of patients with chronic obstructive pulmonary diseases. Probl Tuberk Bolezn Legk.

[16] Michel O. (2006). Nasal irrigation in case of rhinosinusitis. Laryngo Rhino Otol..

[17] Olina M., Aluffi Valletti P., Pia F., Toso A., Borello G., Policarpo M., Garavelli P.L. (2008). Hydrological indications in the therapy of pharyngitis. Recenti Prog. Med..

[18] Passariello A., Di Costanzo M., Terrin G., Iannotti A., Buono P., Balestrieri U., Balestrieri G., Ascione E., Pedata M., Canani F.B. (2012). Crenotherapy modulates the expression of pro-inflammatory cytokines and immunoregulatory peptides in nasal secretions of children with chronic rhinosinusitis. Am. J. Rhinol. Allergy.

[19] Prandelli C., Parola C., Buizza L., Delbarba A., Marziano M., Salvi V., Zacchi V., Memo M., Sozzani S., Calza S. (2013). Sulphurous thermal water increases the release of the anti-inflammatory cytokine IL-10 and modulates antioxidant enzyme activity. Int. J. Immunopathol. Pharmacol..

[20] Kostrzon M., Czarnobilski K., Czarnobilska E. (2015). The influence of pulmonary rehabilitation in the Wieliczka Salt Mine on asthma control-preliminary results. Przegl Lek.

[21] Petraccia L., Masciullo S.G., Grassi M., Pace A., Lucchetta M.C., Valenzi V.I., Avino P., Fraioli A. (2005). Spa and climate therapy in chronic obstructive pulmonary diseases. Clin. Ter..

[22] Chernenkov R.A., Chernenkova E.A., Zhukov G.V. (1997). The use of an artificial microclimate chamber in the treatment of patients with chronic obstructive lung diseases. Vopr Kurortol Fizioter Lech Fiz Kult.

[23] Cantone E., Maione N., Di Rubbo V., Esposito F., Iengo M. (2015). Olfactory performance after crenotherapy in chronic rhinosinusitis in the elderly. Laryngoscope.

[24] Ciprandi G., Cristofolini M., Mira E. (2016). Comano thermal water inhalations in the treatment of allergic rhinitis: Preliminary results. Eur. Ann. Allergy Clin. Immunol..

[25] Costantino M., Giuberti G., Caraglia M., Lombardi A., Misso G., Abbruzzese A., Ciani F., Lampa E. (2009). Possible antioxidant role of SPA therapy with chlorine–sulphur– bicarbonate mineral water. Amino Acids.

[26] Passali D., Gabelli G., Passali G.C., Magnato R., Platzgummer S., Salerni L., Cunsolo S.L., Joos A., Bellussi L.M. (2016). Radioactive Merano SPA treatment for allergic rhinitis therapy. Int. J. Otolaryngol..

[27] Varricchio A., Giuliano M., Capasso M., Del Gaizo D., Ascione E., De Lucia A., Avvisati F., Capuano F., De Rosa G., Di Mauro F. (2013). Salso-sulphide thermal water in the prevention of recurrent respiratory infections in children. Int. J. Immunopathol. Pharmacol..

[28] Kostrzon M., Sliwka A., Wloch T., Szpunar M., Ankowska D., Nowobilski R. (2019). Subterranean pulmonary rehabilitation in chronic obstructive pulmonary disease. Adv. Exp. Med. Biol..

[29] d’Obyrn K., Rajchel L. (2015). Balneoterapeutyczne wykorzystanie solanek w uzdrowisku Kopalnia Soli Wieliczka. Przegl Geol..

[30] Zajac D., Russjan E., Kostrzon M., Kmiecki M., Kaczynska K. (2019). The influence of inhalation of brine from the Wieliczka Salt Mine on inflammation and airway hyperreactivity in a murine model of non-atopic asthma. Eur. Respir. J..

[31] Rubin B.K. (2015). Aerosol medications for treatment of mucus clearance disorders. Respir. Care.

[32] Tanizaki Y., Kitani H., Okazaki M., Mifune T., Mitsunobu F., Honke N. (1993). Clinical effects of complex spa therapy on patients with steroid-dependent intractable asthma (SDIA). Arerugi.

[33] Bar-Yoseph R., Kugelman N., Livnat G., Gur M., Hakim F., Nir V., Bentur L. (2017). Halotherapy as asthma treatment in children: A randomized, controlled, prospective pilot study. Pediatr. Pulmonol..

[34] Maguire C., Cantrill H., Hind D., Bradburn M., Everard M.L. (2015). Hypertonic saline (HS) for acute bronchiolitis: Systematic review and meta-analysis. BMC Pulm. Med..

[35] Daviskas E., Anderson S.D., Gonda I., Eberl S., Meikle S., Seale J.P., Bautovich G. (1996). Inhalation of hypertonic saline aerosol enhances mucociliary clearance in asthmatic and healthy subjects. Eur. Respir. J..

[36] Fujimura M., Amemiya M., Myou S., Mizuguchi M., Matsuda T. (1997). A guinea-pig model of ultrasonically nebulized distilled water-induced bronchoconstriction. Eur. Respir. J..

[37] Kivity S., Poterman R., Schwarz Y., Soferman R., Topilsky M. (1995). Changes in sensitivity to methacholine after inhalation with distilled water: The role of the bronchoconstrictive response. Eur. Respir. J..

[38] Schoeffel R.E., Anderson S.D., Altounyan R.E.C. (1981). Bronchial hyperreactivity in response to inhalation of ultrasonically nebulized solutions of distilled water and saline. Br. Med. J..

[39] Kashiwabara K., Itonaga K., Moroi T. (2003). Airborne water droplets in mist or fog affect nocturnal attacks in asthmatic children. J. Asthma.

[40] Tränkner D., Hahne N., Suginoa K., Hoonb M.A., Zukera C. (2014). Population of sensory neurons essential for asthmatic hyperreactivity of inflamed airways. Proc. Natl. Acad. Sci. USA.

[41] Vanoirbeek J.A., Rinaldi M., De Vooght V., Haenen S., Bobic S., Gayan-Ramirez G., Hoet P.H.M., Verbeken E., Decramer M., Nemery B. (2010). Noninvasive and invasive pulmonary function in mouse models of obstructive and restrictive respiratory diseases. Am. J. Respir. Cell Mol. Biol..

[42] Kraneveld A.D., van der Kleij H.P.M., Kool M., van Houwelingen A.H., Weitenberg A.C.D., Redegeld F.A.M., Nijkamp F.P. (2002). Key role for mast cells in nonatopic asthma. J. Immunol..

[43] Russjan E., Andrzejewski K., Sulejczak D., Kleczkowska P., Kaczyńska K. (2019). Endomorphin-2- and neurotensin- based chimeric peptide attenuates airway inflammation in mouse model of nonallergic asthma. Int. J. Mol. Sci..

[44] Kim H.Y., De Kruyff R.H., Umetsu D.T. (2010). The many paths to asthma: Phenotype shaped by innate and adaptive immunity. Nat. Immunol..

[45] Maltby S., Tay H.L., Yang M., Foster P.S. (2017). Mouse models of severe asthma: Understanding the mechanisms of steroid resistance, tissue remodeling and disease exacerbation. Respirology.

[46] Qin X.-Q., Xiang Y., Liu C., Tan Y.-R., Qu F., Peng L.-H., Zhu X.-L., Qin L. (2007). The role of bronchial epithelial cells in airway hyperresponsiveness. Acta Physiol. Sin..

[47] Boshtam M., Asgary S., Kouhpayeh S., Shariati L., Khanahmad H. (2017). Aptamers against pro- and anti-inflammatory cytokines: A review. Inflammation.

[48] Zaripova T.N., Antipova I.I., Siniagina M.A. (2013). A new approach to the treatment of patients presenting with bronchial asthma and concomitant allergic rhinitis. Vopr Kurortol Fizioter Lech Fiz Kult.

[49] Yokoyama A., Kohno N., Fujino S., Hamada H., Inoue Y., Fujioka S., Ishida S., Hiwada K. (1995). Circulating interleukin-6 levels in patients with bronchial asthma. Am. J. Respir. Crit. Care Med..

[50] Liao Z., Xiao H., Zhang Y., Tong R.-S., Zhang L.-J., Bian Y., He X. (2005). IL-1beta: A key modulator in asthmatic airway smooth muscle hyper-reactivity. Expert Rev. Respir. Med..

[51] Tsukagoshi H., Sakamoto T., Xu W., Barnes P., Chung F. (1994). Effect of interleukin-1β on airway hyperresponsiveness and inflammation in sensitized and nonsensitized Brown-Norway rats. J. Allergy Clin. Immunol..

[52] Barnes T.C., Anderson M.E., Moots R.J. (2011). The Many Faces of Interleukin-6: The Role of IL-6 in Inflammation, Vasculopathy, and Fibrosis in Systemic Sclerosis. Int. J. Rheumat..

[53] Johnson V.J., Yucesoy B., Luster M.I. (2005). Prevention of IL-1 signaling attenuates airway hyperresponsiveness and inflammation in a murine model of toluene diisocyanate–induced asthma. J. Allergy Clin. Immunol..

[54] Ray A., Kolls J.K. (2017). Neutrophilic inflammation in asthma and association with disease severity. Trends Immunol..

[55] Krane C.M., Fortner C.N., Hand A.R., McGraw D.W., Lorenz J.N., Wert S.E., Towne J.E., Paul R.J., Whitsett J.A., Menon A.G. (2001). Aquaporin 5-deficient mouse lungs are hyperresponsive to cholinergic stimulation. Proc. Natl. Acad. Sci. USA.

[56] Hoffert J.D., Leitch V., Agre P., King L.S. (2000). Hypertonic Induction of Aquaporin-5 Expression through an ERK-dependent Pathway. J. Biol. Chem..

[57] Nilsson H., Dragomir A., Ahlander A., Johannesson M., Roomans G.M. (2007). Effects of hyperosmotic stress on cultured airway epithelial cells. Cell Tissue Res..

[58] Wark P.A., Donald V.M., Jones A.P. (2005). Nebulized hypertonic saline for cystic fibrosis. Cochrane Database Syst. Rev..

[59] Comhair A., Erzurum S.C. (2010). Redox control of asthma: Molecular mechanisms and therapeutic opportunities. Antioxid Redox Signal.

[60] Fitzpatrick A.M., Teague W.G., Holguin F., Yeh M., Brown L.A.S. (2009). for the Severe Asthma Research Program. Airway glutathione homeostasis is altered in children with severe asthma: Evidence for oxidant stress. J. Allergy Clin. Immunol..

[61] Smith L.J., Houston M., Anderson J. (1993). Increased levels of glutathione in bronchoalveolar lavage fluid from patients with asthma. Am. Rev. Respir. Dis..

[62] Kleniewska P., Pawliczak R. (2017). The participation of oxidative stress in the pathogenesis of bronchial asthma. Biomed Pharm..

[63] Wohlauer M., Moore E.E., Silliman C.C., Fragoso M., Gamboni F., Harr J., Accurso F., Wright F., Haenel J., Fullerton D. (2012). Nebulized hypertonic saline attenuates acute lung injury following trauma and hemorrhagic shock. Crit. Care Med..

[64] Wright F.L., Gamboni F., Moore E.E., Nydam T.L., Mitra S., Silliman C.C., Banerjee A. (2014). Hyperosmolarity invokes distinct anti-inflammatory mechanisms in pulmonary epithelial cells: Evidence from signaling and transcription layers. PLoS ONE.

[65] Barbieri M., Salami A., Mora F., Casazza A., Sovatzis A., Teglia R., Cordone M.P., Mora R. (2002). Behavior of serum IgE and IgA in patients with allergic rhinitis treated with iodine bromide thermal water. Acta Otorhinolaryngol. Ital..

[66] Bellometti S., Bertocco E., Galzigna L. (1998). Changes in the intrabronchial microflora of patients with chronic bronchitis after inhaling mineral water. Vopr Kurortol Fizioter Lech Fiz Kult.

[67] Matveeva L.A., Kuzmenko O.V., Kurts I.A., Golosova L.O. (1998). The use of an aerosol of natural brine and of sinusoidal modulated currents in chronic pneumonias in children. Vopr Kurortol Fizioter Lech Fiz Kult.

[68] Keller S., König V., Mösges R. (2014). Thermal water applications in the treatment of upper respiratory tract diseases: A systematic review and meta-analysis. J. Allergy.

[69] Viegas J., Esteves A.F., Cardoso E.M., Arosa F.A., Vitale M., Taborda-Barata L. (2019). Biological effects of thermal water-associated hydrogen sulfide on human airways and associated immune cells: Implications for respiratory diseases. Front. Public Health.

[70] de Baaij J.H.F., Hoenderop J.G.J., Bindels R.J.M. (2015). Magnesium in man: Implications for health and disease. Physiol. Rev..

[71] Emelyanov A., Fedoseev G., Barnes P.J. (1999). Reduced intracellular magnesium concentrations in asthmatic patients. Eur. Respir. J..

[72] Hashimoto Y., Nishimura Y., Maeda H., Yokoyama M. (2000). Assessment of magnesium status in patients with bronchial asthma. J. Asthma.

[73] Gilliland F.D., Berhane K.T., Li Y.F., Kim D.H., Margolis H.G. (2002). Dietary magnesium, potassium, sodium, and children’s lung function. Am. J. Epidemiol..

[74] Hirota N., Martin J.G. (2013). Mechanisms of airway remodeling. Chest.

[75] Okayama H., Aikawa T., Okayama M., Sasaki H., Mue S., Takishima T. (1987). Bronchodilating Effect of intravenous magnesium sulfate in bronchial asthma. JAMA.

[76] Powell C.V.E., Kolamunnage-Dona R., Lowe J., Boland A., Petrou S., Doull I., Hood K., Williamson P.R. (2013). MAGNETIC study group. MAGNEsium Trial in Children (MAGNETIC): A randomised, placebo-controlled trial and economic evaluation of nebulised magnesium sulphate in acute severe asthma in children. Health Technol. Assess..

[77] Nakaya M., Dohi M., Okunishi K., Nakagome K., Tanaka R., Imamura M., Baba S., Takeuchi N., Yamamoto K., Kaga K. (2006). Noninvasive system for evaluating allergen-induced nasal hypersensitivity in murine allergic rhinitis. Lab. Investig..

[78] Niu C., Wang T., Zou W., Hu J., Ying L., Zhang M., Liu J., Tian D., Dai J., Luo Z. (2019). Enhanced pause correlates with airway neutrophils and airway-epithelial injury in asthmatic mice treated with dexamethasone. J. Asthma.

[79] Vanoirbeek J.A., Tarkowski M., De Vooght V., Nemery B., Hoet P.H. (2009). Immunological determinants in a mouse model of chemical-induced asthma after multiple exposures. Scand. J. Immunol..

[80] Lundblad L.K.A., Irvin C.G., Hantos Z., Sly P., Mitzner W., Bates J.H.T. (2007). Penh is not a measure of airway resistance!. Eur. Respir. J..

[81] Lundblad L.K.A., Irvin C.G., Adler A., Bates J.H.T. (2002). A reevaluation of the validity of unrestrained plethysmography in mice. J. Appl. Physiol..

[82] Zhang Q., Lai K., Xie J., Chen G., Zhong N. (2009). Does unrestrained single-chamber plethysmography provide a valid assessment of airway responsiveness in allergic BALB/c mice?. Respir. Res..

[83] Hamelmann E., Schwarze J., Takeda K., Oshiba A., Larsen G.L., Irvin C.G., Gelfand E.W. (1997). Noninvasive measurement of airway responsiveness in allergic mice using barometric plethysmography. Am. J. Respir. Crit. Care Med..

[84] Adler A., Cieslewicz G., Irvin C.G. (2004). Unrestrained plethysmography is an unreliable measure of airway responsiveness in BALB/c and C57BL/6 mice. J. Appl. Physiol..

[85] Duguet A., Biyah K., Minshall E., Gomes R., Wang C.-G., Taoudi-Benchekroun M., Bates J.H.T., Eidelman D.H. (2000). Bronchial responsiveness among inbred mouse strains. Role of airway smooth-muscle shortening velocity. Am. J. Respir. Crit. Care Med..

[86] van der Kleij H.P.M., Kraneveld A.D., van Houwelingen A.H., Kool M., Weitenberg A.C.D., Redegeld F.A.M., Nijkamp F.P. (2004). Murine model for non-IgE-mediated asthma. Inflammation.

[87] Russjan E., Kaczyńska K. (2019). Beneficial effects of neurotensin in murine model of hapten-induced asthma. Int. J. Mol. Sci..

[88] Russjan E., Kaczyńska K. (2018). Murine models of hapten-induced asthma. Toxicology.

